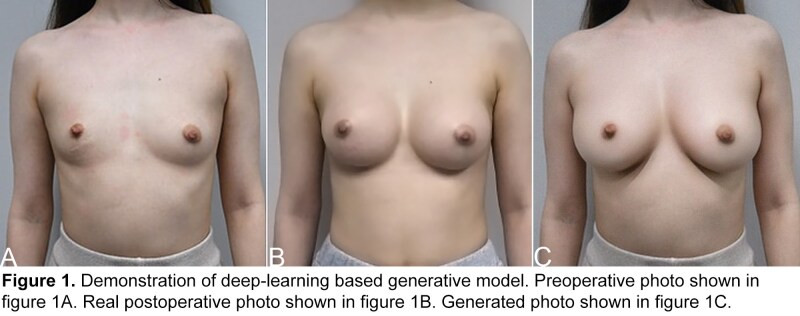# Augmentation AI: Use of a Novel Deep Learning-Based Generative Artificial Intelligence System As a Tool for Breast Augmentation Simulation

**DOI:** 10.1093/asjof/ojaf018.019

**Published:** 2025-05-13

**Authors:** Waylon Zeng, Andrew Schroeder, Kurtis Moyer, James Thompson

**Affiliations:** Private Practice, Roanoke, VA; Private Practice, Roanoke, VA; Private Practice, Roanoke, VA; Private Practice, Roanoke, VA

## Abstract

**Goals/Purpose:**

The purpose of this study is to evaluate the clinical efficacy of a custom deep learning-based image generating system for preoperative breast augmentation simulation by evaluating plastic surgeons' ability to distinguish between real postoperative photographs and AI-generated simulations.

**Methods/Technique:**

A retrospective analysis was conducted using preoperative photographs from 30 patients who underwent bilateral breast augmentation between 2015 and 2024. A novel three-stage image generation pipeline was developed utilizing a custom-trained Stable Diffusion AI model. The first stage involved an image segmentation model to identify and separate the nipple. Then a depth estimation model was created to create a depth map from two-dimensional preoperative photographs to capture three-dimensional breast morphology. The depth maps and nipple segmentation models then guided the third stage, where the Stable Diffusion model generated predicted postoperative results while maintaining anatomical accuracy and patient-specific features. Fifteen cases were processed through the AI system, while fifteen matched cases served as controls using actual postoperative photographs. A panel of 15 plastic surgeons, comprising 5 board-certified attending surgeons and 10 plastic surgery residents, evaluated the images, viewing preoperative photographs followed by either real postoperative photographs or AI-generated simulations. Evaluators were asked to classify each postoperative image as either real or AI-generated. Statistical analysis employed a two-tailed binomial test comparing the proportion of correct classifications against random chance (50%), with significance set at p < 0.05.

**Results/Complications:**

Analysis of 450 total evaluations (15 surgeons × 30 cases) demonstrated that panelists correctly identified the origin of postoperative images in 234 cases (52.0%). The binomial test revealed no statistically significant difference between the observed correct guess rate and random chance (p = 0.57, 95% CI: 47.3%-56.7%). Subgroup analysis showed similar accuracy rates between attending surgeons (53.3% correct, p = 0.51) and residents (51.3% correct, p = 0.64). No significant difference was observed in guess accuracy between real postoperative photographs (52.4% correct, p = 0.46) and AI-generated simulations (51.6% correct, p = 0.53).

**Conclusion:**

This study demonstrates that our deep learning-based breast augmentation simulation system produces photorealistic results indistinguishable from actual postoperative photographs by experienced plastic surgeons and residents in training. The three-stage pipeline incorporating nipple segmentation and depth map analysis demonstrates reliable anatomical accuracy during image generation. These findings suggest that AI-generated simulations may serve as a reliable tool for preoperative planning and patient communication. Future studies should assess the system's predictive accuracy by limiting human interaction with determining the best image and evaluate its impact on patient satisfaction and surgical decision-making.

**Figure:**